# Frontline nurses’ adherence to COVID-19 policies in care delivery at a Johannesburg Academic Hospital

**DOI:** 10.4102/curationis.v48i1.2685

**Published:** 2025-05-09

**Authors:** Fhulufhelo Mulaudzi, Charlene Downing

**Affiliations:** 1 Department of Nursing, Faculty of Health Science, University of Johannesburg, Johannesburg, South Africa

**Keywords:** coronavirus disease (COVID-19), policies, frontline nurses, nurses, academic hospital

## Abstract

**Background:**

Nurses’ adherence to coronavirus disease 2019 (COVID-19) policies significantly impacts infection rates, yet various factors, including communication gaps, lack of policy involvement and insufficient training, hinder compliance.

**Objectives:**

This study investigated the availability of COVID-19 infection control policies and frontline nurses’ adherence to these policies at an academic hospital in Johannesburg.

**Method:**

A quantitative, descriptive-comparative design was employed, using stratified random sampling across three phases: policy document analysis, adherence assessment and observation. Phase one evaluated the comprehensiveness of policies from National Institute for Communicable Diseases (NICD), Department of Health (DoH) (SA), National Institutes of Health (NIH) and Centers ffor Disease Control and Prevention (CDC).

**Results:**

National Institute for Communicable Diseases, DoH and academic hospital policies were 100% adequate, while CDC and NIH policies met 82% of assessed attributes. Phases two and three assessed adherence using questionnaires and observations. Results revealed higher adherence during direct patient contact (median 5/7; 70%) compared to pre- and post-contact behaviours (median 3/6; 50%). Principal component analysis showed a strong correlation (*r* = 0.903) between adherence and general precautionary measures.

**Conclusion:**

Despite partial compliance, findings highlight a need for enhanced in-service training and improved communication strategies to promote policy adherence and minimise infection risks.

**Contribution:**

Recommendations are provided to strengthen nursing practice, education and policy development, empowering nurses with knowledge and strategies for effective infection control.

## Introduction

In 2019, a corona virus disease (COVID-19), caused by a respiratory-affecting virus, emerged in China and rapidly spread across the globe and had an impact on nurses’ functioning. High infection and mortality rates were recorded among healthcare professionals. The World Health Organization (WHO 2020) deemed the outbreak a global pandemic (Raoofi et al. 2020:220). Frontline nurses globally faced the task of saving lives and informing themselves and the public on how best to protect themselves. However, these nurses were impacted by lack of resources including staff shortage, a shortage of personal protective equipment (PPE) and conflicting values (Turale, Meechamnan & Kunaviktikul 2020:165). The pandemic severely impacted frontline nurses as they were dealing with very diverse situations in rendering care and a lack of skills and knowledge contributed to nurses’ distress during this difficult time (Rathnayake et al. 2021:1). Frontline nurses rendered care to COVID-19 patients without special skills and knowledge, which included but were not limited to special training on caring for COVID-19 patients, training on donning and doffing posing a severe risk to their own health (Rathnayake et al. 2021:1). Rathnayake et al. (2021:1) also reported frontline nurses identified insufficient policy development to direct functioning and infection prevention while dealing with COVID-19 patients.

Coronavirus disease 2019 policies are a set of rules designed to influence change or contain a problem (Mintrom & O’Connor 2020:1). Policies are developed to guide the thinking and functioning of a larger population (Mintrom & O’Connor 2020:1). Nurses ultimately look up to their managers and stakeholders to provide them with sufficient resources and relevant information on safe practice standards during crises (Fernandez et al. 2020:1). Thus, as available policies and guidelines may not apply to the pandemic, new protocols had to be established, given a change in work dynamics (Carter & Notter 2020:5). Literature suggests that policies, protocols and guidelines were established on measures to be enforced while caring for COVID-19 patients (Al-Jabir et al. 2020:169). Kovner (2020:2) further emphasises that if policymakers and healthcare professionals invested their resources in ensuring the functionality of public health, numerous lives would have been saved. It is necessary to continuously design nursing learning programmes to empower nurses, as the lack of these programmes may contribute to poor adherence to COVID-19 policies (Daly et al. 2020:2752).

A study indicated high adherence to Infection and Prevention Control (IPC) practice during COVID-19 and it has proven to be effective, and a reduced rate of infection was recorded (Darko et al. 2024:5). Knowledge has contributed highly on the adherence; however, this study indicated a need for more training and knowledge as respondents obtained below 40% for waste segregation (Darko et al. 2024:6). Globally, hospitals have reported different challenges in caring for COVID-19 patients and ensuring staff is safe (Neil 2020:273). As of September 2020, South Africa employed 13 135 nurses to cater to the pandemic, but the infection rate remained high, with 31 000 healthcare workers being infected; most were nurses and midwives (South African Government News Agency 2020). Leaders faced challenges in mitigation strategies, testing, staffing, supplies and acquiring durable equipment (Neil 2020:273). Moreover, PPE guidelines did not prevent nurses from reusing supplies even after intense training was provided on proper use because of shortages (Neil 2020:273). This puts nurses at a high risk of contracting the virus. The study aimed to investigate the availability of COVID-19 infection control policies and frontline nurses’ adherence to these policies during the Covid pandemic at an academic hospital. The study’s outcome will assist in identifying gaps, and recommendations will enable or encourage nursing departments to re-align themselves. In addition, the outcomes of the study could also be used to facilitate recommendations on future disaster preparedness and clinical curriculum development, including disaster planning development and application.

## Research methods and design

### Study design and samples

The study selected organisational documents for desk analysis, and all documents were compliant and had all required attributes, except those of the National Institutes of Health (NIH 2021) and the Centers for Disease Control and Prevention (CDC). The second phase of the study was conducted with 194 nurses and phase three with 46 nurses. Respondents were between 20 and 61 years and older. The study was conducted in an academic hospital in Johannesburg, Gauteng, South Africa. The hospital had a capacity of 3400 beds with different specialities and catering to diverse health professionals learning. The professional and support staff exceeds 6760 people. The staff and patient ratio was not stable, and staff shortages have always been a problem in the nursing profession. The study was conducted in emergency departments rendering services to all patients, either with an unknown COVID-19 status, a negative or a positive status. These departments included the casualty department as the receiving ward for all patients, the operating theatre as an emergency department for those who needed emergency operations and the intensive care unit (ICU), as an emergency department for the critically ill. These departments were chosen after an analysis of the literature reflected where most COVID-19 cases were admitted, according to the documents the researcher assessed (Quah et al. 2020:1). These departments were chosen because they offered 24-h emergency care, they were continually receiving new patients and the researcher believes these department were highly saturated with COVID-19 patients and an area of interest for this study.

The study was conducted over three phases: phase one was a document analysis comparing policies against five guidelines developed for COVID-19, phase two was an analysis of nurses’ adherence to COVID-19 protocols, phase three was an observation carried out as nurses rendered nursing care. During phase one of the study, the researcher reviewed government and organisational guideline documents relating to COVID-19, published from the onset of the pandemic in March 2020 until March 2021. These documents were retrieved from the COVID-19 database website, and thus phase one was a desk analysis. A quantitative research design, descriptive comparative and cross-sectional research method were used for this study to delineate variables and note the difference presented among different groups. The above-mentioned design and method were applied in phase two and three using the stratified random sampling.

A sample of 476 nurses across different disciplines was available. The accessible population included *n* = 271 professional nurses (theatre *n* = 104, ICU *n* = 99 and casualty *n* = 68), *n* = 101 staff nurses (theatre *n* = 53, casualty *n* = 35 and ICU *n* = 13) and *n* = 104 enrolled nursing assistants (casualty *n* = 53, theatre *n* = 45 and ICU *n* = 6). The reason for 271 accessible population was because of different reasons, different work times (off duties), some on different leave days, etc. A total of *n* = 213 nurses comprised the predetermined sample using the Raosoft method. However, 220 questionnaires were distributed in phase two and only 194 returned the questionnaires, and thus data were collected from 194 nurses in this phase. The acceptance rate for the study was 88% of nurses rendering care to COVID-19 patients. Based on the response rate, the study’s findings can be generalised and applied in future studies. Phase three sampling size was determined by generating 20% of the main sample, and the total was 39–40 respondents. During phase three, 50 information letters and consent forms were distributed, and only 46 completed consent forms were returned to the researcher. The response rate for this phase was 92%, which was a good response. The study inclusion criteria adopted for the study was as follows: respondents needed to be 18 years or older, a nurse working in the frontline and five emergency departments rendering nursing care during the COVID-19 pandemic and willing to complete the structured questionnaire.

### Data-collection tools

#### Coronavirus disease 2019 policy analysis

Phase one consisted of a document analysis; the researcher intended to determine whether policies designed to care for patients during COVID-19 were available and appropriate in caring for these patients. The aim was to compare whether the policies were aligned with the WHO Infection prevention and control strategies designed to ensure a decrease in the spread of COVID-19 and channel uniform practice that will enable adherence among healthcare workers. The controls included environmental and administrative strategies, the use of PPE, staff training on COVID-19 guidelines, waste management and corpse handling. Document analysis entailed getting the material ready, extracting data, analysing data and distilling the findings (Dalglish, Khalid & McMahon 2020:1424). Kayesa and Shung-King (2020:2) claim that document analysis is a process that involves an in-depth study of document contents to report on and interpret findings.

Authors compiled government and organisational guideline documents relating to COVID-19 from the onset of the pandemic in March 2020 until March 2021. The following keywords were applied: ‘COVID-19’, ‘policies’, ‘nursing protocols and guidelines’, ‘infection control’ and ‘adherence’. Documents accessed for this phase included the COVID-19 guiding documents from the Centers for Disease Control and Prevention (CDC), academic hospital policy document, Department of Health (DOH), National Institute for Communicable Diseases (NICD 2021), NIH and WHO. Authors developed a data-collecting sheet to reflect on the attributes of policy evaluation. The data-collecting sheet was assessed for the availability of the following attributes in the identified documents for the training of staff on COVID-19 guidelines; hand hygiene, environmental cleaning and disinfection, linen disposal, waste management, proper use of PPE, donning and doffing, isolation room principle, restricted access signage and dead bodies disposal. A scoring method was developed after consulting with a statistician, and a point was awarded for each identified attribute in the policy documents. The analysis explored alignment in terms of the sequence, correctness (Miles 2019:2) and strength of policy documents, which reflected the documents’ strength in terms of covering all aspects of caring for COVID-19 patients (Miglianico et al. 2019:6).

#### Socio-demographic

Respondents’ biographic details were obtained from the self-administered hard-copy questionnaire with the following attributes: respondents’ age, gender, marital status, ethnicity, highest qualification, professional rank, employment status, area of employment, years of experience, indication if nurses rendered nursing care to suspected or confirmed COVID-19 patients and the number of patients a nurse rendered care to in a week.

#### Coronavirus disease 2019 policy adherence scale

The COVID-19 policy adherence scale was developed to measure nurses’ adherence to the hospital’s COVID-19 policy. The scale was designed from a guiding document from the WHO, and permission to use or reproduce the tool was obtained (WHO, IPC Guidelines 2021). The COVID-19 policy adherence instrument consisted of 14 questions that were answered against a five-point Likert scale. The instrument was deemed reliable and showed great internal consistency with Cronbach’s alpha of 0.852. A pilot study was conducted before the main study, and during this process, instrument validity was confirmed. Twenty questionnaires were distributed in two departments, an ICU and emergency casualty unit for pilot study. In total, 16 completed questionnaires were collected. The acceptance rate of the study was 80%.

#### Coronavirus disease 2019 observation scale

The observation scale consisted of 14 questions similar to the COVID-19 policy adherence scale adopted from the WHO (WHO, IPC Guidelines 2021) and the South African Department of Health (DoH 2021). This tool was used to assess whether nurses rendered care to suspected or confirmed COVID-19 cases, if nurses made use of N95 masks, gloves, gowns, face shields and waterproof aprons, if an appropriate hand washing technique was practiced, proper use of PPE, proper disposal of PPE, decontamination of surfaces and use of COVID-19 signage (WHO, IPC Guidelines 2021). Face and construct validity was confirmed through a pilot study and through measuring of adherence variables on data analysis.

### Data collection

Data collection occurred in phases, phase one’s data were collected on a data-collection instrument. It began with a compilation of relevant research on the study’s topic; then, data were assessed to draw a conclusion on the findings (Ahn & Kang 2018:103). Authors developed an abstraction tool by searching relevant websites for current data recorded on COVID-19 guidelines. Authors compiled government and organisational guideline documents relating to COVID-19 from the onset of the pandemic in March 2020 until March 2021. Documents included soft and hard copies that were prepared for analysis purposes. A data-collecting sheet was used for policy document scoring.

Phase two spanned a period from 11 March 2022 to 28 March 2022. Data were collected at different time intervals (day shift and night shift) as this afforded everyone in the research population an opportunity to participate in the study. Phase two’s data were collected using hard-copy questionnaires that included an information letter and consent form. Phase two began with respondents assessing for policy evaluation, and this exercise aimed to identify whether the policy attributes were aligned with the WHO COVID-19 guidelines within the hospital. For each question on the policy and attributes questionnaire, one point was awarded for ‘yes’ and zero for ‘no’. The COVID-19 policy adherence instrument consisted of 14 self-administered questionnaire against a five-point Likert scale.

Phase three’s data were collected by observing a sample of 46 nurses (*n* = 20 professional nurses, *n* = 12 staff nurses and *n* = 14 auxiliary nurses) from 14 April 2022 until 17 April 2022. Before phase three’s data collection, an information letter and consent form were distributed and explained to the respondents, and consent was obtained. The same principles from phase two were applied for data collection, and the respondents signed the consent form on 13 April 2022, before the observation assessment could begin. The researcher ensured the data collection’s accuracy by obtaining consent separately for each phase and later randomly observed nurses while they carried out their duties. This ensures that respondents were not aware of the observation process, and it was conducted for the duration of the procedure to mitigate bias.

### Data analysis

A deductive approach was used to determine whether policies contained adequate information, as per the WHO guidelines, making use of the designed proforma (Raoofi et al. 2021:261). A point was awarded for each identified attribute. Thereafter, a total was generated by calculating the awarded points. A proforma analysis was conducted against the developed results to determine adequacy, and comparisons were made to draw conclusions. Documents with all 11 attributes were deemed completely adequate, 10-8 attributes were partially adequate and 7-0 were inadequate (Raoofi et al. 2021:261). Data from phases two and three were analysed through content analysis and descriptive statistics, such as frequencies and percentages (Gray, Grove & Sutherland 2017:363). Similar responses were grouped together so that the percentage of similar responses could be calculated. Numeric codes were assigned to these groups, in accordance with the most common value in a set of values. Data were expressed in percentage form, and coding was further employed to identify the highest and lowest values in relation to specific questions in the study (Shepeard 2019:1–4). Data were also analysed using Statistical Package for Social Science software version 27, (SPSS), and a comparison analysis was performed to attain correlation and factor analysis (Sullivan-Bolyai & Bova 2014:310). The one-way analysis of variance was also applied; variables were evaluated to confirm normality distribution using the Kolmogorov–Smirnov test. This study’s diagnostic test reflected a value of 0.812, which was a good indication that factor analysis would be of great use to the study. Bartlett’s test of sphericity was also conducted to determine the correlation matrix, and this value had to be greater than 0.5. The pattern matrix method was applied to group factors in three groups. Principal component analysis (PCA) extraction and rotational methods (Oblimin with Kaiser Normalisation) were also used.

### Ethical considerations

Prior to commencing the research, ethical clearance was obtained from the Research Ethics Committee (reference no.: REC-1176- 2021) and Higher Degrees Committee (reference no.: HDC-01-72-2021) Permission was also granted by an academic hospital and the hospital’s ethics committee to conduct the study in their facilities. The academic hospital is affiliated with a university, and a further human research clearance certificate was obtained from this university (reference no.: M211008). Principles of autonomy, beneficence, non-maleficence and justice were adhered to throughout the study. Respondents received an information letter, accompanied by a verbal explanation of the study’s details, and all respondents provided written consent before they were included in the study.

## Results

The results are tabulated through descriptive and inferential statistical analysis. In phase one, the WHO, National Institute for Communicable Diseases (NICD), DoH (SA) and an academic hospital in Johannesburg had all 11 attributes, and documents were thus deemed completely adequate. The Centers for Disease Control and Prevention (CDC) and NIH reflected nine of the 11 attributes, which indicated 82% of the attributes were included by each of the two organisations.

Phase two, policy evaluation scoring indicated a mean value of 5.97 and a standard deviation of 2.587. A normality test was also conducted to confirm the policy evaluation results using the Kolmogorov–Smirnov testing method. The results indicated that the sample did not have a normal distribution because most nurses scored the institution’s COVID-19 policy high; very few scored the policy low. The policy attributes’ scoring reflected a greater number of respondents agreed the COVID-19 policy had all the required attributes, 124 (63.9%). This was followed by an attribute scoring of nine out of 10 (*n* = 15; 7.7%), 8 (*n* = 14; 7.2%), 7 (*n* = 5; 7%), 6 (*n* = 6; 3.6%), 5 (*n* = 7; 3.6%), 4 (*n* = 6; 3.1%), 3 (*n* = 4; 2.1%), 2 (*n* = 1; 0.5%) and 0 (*n* = 4; 2.1%). Phase two adopted a total variance extraction tool validates factor analysis in this study. The factors in this study were extracted using the PCA method, and three factors were extracted and tabulated further in the text (Tavakol & Wetzel 2020:245).

### Adherence factor 1 – General coronavirus disease 2019 protocols adopted by nurses in rendering care to suspected or confirmed coronavirus disease 2019 patients

The cumulative percentage of the three factors of adherence indicated a value of 59.337%, which is acceptable for factor analysis. Moreover, the pattern matrix method was applied to group factors in three groups. Principal component analysis extraction and rotational methods (Oblimin with Kaiser Normalisation) were also employed. The rotation converged into 13 iterations, which are displayed further in the text. Factor 1 consisted of six components of the research questionnaire, namely c13, c10, c14, c12, c11 and c6, and all questions addressed general protocols nurses would practice when rendering nursing care to COVID-19 patients. The factors are displayed in Table 1.

**TABLE 1:** Factor analysis results.

Factor	Item number	Item description	Item value	Item value	Item value
**1 – General COVID-19 protocols adopted by nurses in rendering care to suspected or confirmed COVID-19 patients. (All nursing categories used in the study).**					
	c13	Do you use a proper solution for hand washing, cleaning COVID-19 surfaces and disinfecting reusable personal protective equipment (PPE)?	0.783	−0.037	0.051
	c10	During healthcare interactions with COVID-19 patients, were all surfaces and high surfaces decontaminated?	0.770	−0.042	−0.128
	c14	Do you use ‘no entry’ signage for suspected or confirmed COVID-19 patients?	0.731	−0.055	−0.218
	c12	Do you use proper disposal techniques for all supplies used for suspected or confirmed COVID-19 patients?	0.720	0.086	0.337
	c11	Do you use proper donning and doffing techniques before and after contact with suspected or confirmed COVID-19 patients? Do you have an assistant when donning and doffing?	0.713	0.022	0.306
	c6	Do you wear a face shield before you are in contact with a suspected or confirmed COVID-19 patient?	0.486	−0.222	0.148
**2 – Direct protocols adopted by nurses in rendering care to suspected or confirmed COVID-19 patients. (All nursing categories used in the study).**					
	c8	Do you practice hand hygiene before and after being in contact with suspected or confirmed COVID-19 patients?	0.059	−0.819	−0.253
	c9	Do you perform hand hygiene after touching the COVID-19 surroundings (bed, door handles)?	0.027	−0.807	−0.221
	c2	Do you wear an N95 mask or respirator when entering a room with a suspected or confirmed COVID-19 patient?	−0.192	−0.777	0.323
	c3	Do you make sure the mask and the respirator are a perfect fit with a seal before being in contact with suspected or confirmed COVID-19 patients?	−0.120	−0.670	0.406
	c4	Do you wear gloves before you come in contact with a suspected or confirmed COVID-19 patient?	0.114	−0.641	−0.027
	c5	Do you wear a gown before you come in contact with a suspected or confirmed COVID-19 patient?	0.158	−0.621	−0.041
	c7	Do you wear a waterproof apron during aerosol-generating procedures of suspected or confirmed COVID-19 patients?	0.242	−0.489	0.276

*Source:* Adapted from, Muluadzi, F., 2022, ‘Frontline Nurses’ Adherance to COVID-19 Policies in Rendering Nursing Care during the Pandemic in an Academic Hospital in Johannesburg’, unpublished Masters thesis, University of Johannesburg, Johannesburg

COVID-19, coronavirus disease 2019; c, component.

### Adherence factor 2 – Direct protocols adopted by nurses in rendering care to suspected or confirmed coronavirus disease 2019 patients

Factor 2 addressed direct protocols nurses adopted in rendering care to suspected or confirmed COVID-19 patients. This factor consisted of questions c8, c9, c2, c3, c4, c5 and c7 (see Table 1).

### Adherence factor 3 – Do nurses render care to suspected or confirmed coronavirus disease 2019 patients?

The third factor the researcher identified had one component and/or question (c1) to indicate whether nurses were rendering nursing care to suspected or confirmed COVID-19 patients. This component was identified and extracted because it had a significant value of 0.706.

In this study, correlations were identified and reflected based on different strengths, which are large, medium, small correlations and no correlations. A large correlation was shown as follows: a higher correlation score (*r* = 0.903) was noted between the variable adherence and factor 1 (general precautions), the second highest correlation (*r* = 0.775) was reported between adherence and factor 2 (direct contact precautions), followed by the correlation between the policy score and policy attributes with a value of (*r* = 0.652), the correlation between policy attributes and adherence factor 1 (*r* = 0.561) and lastly, the correlation between policy attribute and adherence score (*r* = 0.534). Medium correlations were identified between policy score and adherence factor 1 (*r* = 0.489), between adherence score and factor 3 (aspect on whether nurses render nursing care to suspected or confirmed COVID-19 patients) (*r* = 0.489), between adherence factor 1 and adherence factor 2 (*r* = 0.450), between policy score and adherence score (*r* = 0.404) and between adherence factor 1 and factor 3 (*r* = 0.375).

Small correlations were identified on the following: policy attributes and adherence factor 2 (*r* = 0.299), policy attribute and factor 3 (*r* = 0.260), adherence factor 2 and factor 3 (*r* = 0.252), policy factor and factor 3 (*r* = 0.168) and policy factor and adherence factor 2 (*r* = 0.141). All correlations were identified as significant and valid in all relationships, as the correlation indicated a value (*p* = 0.000) for all attributes. The correlation indicated positive relationships among the identified variables, as the value was less than 0.05.

In phase three, respondents acquired a 100% rating on assessment on availability of supplies. The required supplies included: N95 masks, gloves, gowns, face shields, waterproof aprons, hand washing solution, decontamination solutions, waste disposal containers, head covers and COVID-19 signage. Good rate of adherence was identified during the observation phase, with a mean of 9.35 (of over 14); however, some respondents attained a negative score for not performing a required adherence act with 14 respondents below average of the 46 respondents The results indicated a maximum score of 14 (six respondents) and a minimum score of 4 (three respondents) in the observation phase. None of the respondents scored a zero, and a good mean scoring was indicated, with an overall score of 9.35 from all respondents.

Phase three results were further illustrated using factor analysis. Factor 1 focused on pre- or post-contact scores. The scoring in this factor indicated high adherence. Adherence factor 2 addressed questions that dealt with direct contact with the patient, and adherence was higher for contact items than for pre- or post-contact items. The median was 3/6 (50% adherence) for pre- or post-contact behaviours and 5/7 (70% adherence) for contact behaviours. Higher significance was generated from adherence factor 2 (*p* = 0.000), indicating that nurses adhered more to contact measures. The second highest significance was generated from adherence factor 1 (*p* = 0.002), and the lowest significance was on adherence score (*p* = 0.031). The results illustrated that respondents rated their adherence as being high, but the researcher’s observation scores suggested otherwise.

### Pearson correlation

Pearson correlation is applied to determine the degree of correlation in a specific measuring description, called correlation coefficient (Yadav 2018:116). The correlations in phase two of the study (adherence) were indicated based on different strengths, namely large, medium, small correlations and no correlations. The highest correlation score (*r* = 0.903) was between the variable adherence and factor 1 (general precautions); the second highest correlation (*r* = 0.775) was between adherence and factor 2 (direct contact precautions); followed by the correlation between the policy score and policy attributes, with a value of *r* = 0.652; the correlation between policy attributes and adherence factor 1 (*r* = 0.561) and lastly, the correlation between policy attribute and adherence score (*r* = 0.534). Medium correlations were reported between policy score and adherence factor 1 (*r* = 0.489); between adherence score and factor 3 (aspect on whether nurses render nursing care to suspected or confirmed COVID-19 patients) (*r* = 0.489); between adherence factor 1 and adherence factor 2 (*r* = 0.450); between policy score and adherence score (*r* = 0.404) and between adherence factor 1 and factor 3 (*r* = 0.375). Small correlations were identified between policy attributes and adherence factor 2 (*r* = 0.299); policy attribute and factor 3 (*r* = 0.260); adherence factor 2 and factor 3 (*r* = 0.252); policy factor and factor 3 (*r* = 0.168) and policy factor and adherence factor 2 (*r* = 0.141). All correlations were significant and valid in all relationships, as the correlation indicated a value of *p* = 0.000 for all attributes. The correlation reflected positive relationships among the identified variables, as the value was less than 0.05.

## Discussion

### Coronavirus disease 2019 policy

The outbreak of COVID-19 caused global stress, and lives were lost because of a lack of information and fear while nurses had to care for infected patients (Silverman et al. 2021:1). Silverman et al. (2021:1) identified some gaps in the unavailability of Covid-related information, risks associated with the job, lack of communication and a lack of functioning policies. With relevant information and adequate training on policies, much can be achieved as nurses will know what procedures to perform and how. Policies eradicate any confusion and create uniformity in standardised guidelines, as stipulated by the WHO; adherence is therefore encouraged (Abdulah, Mohammedsadiq & Liamputtong 2021:296).

A lack of information and directive communication significantly affects how employees behave. In this study, most respondents agreed a relevant COVID-19 policy was available in their institution, but fewer offered positive responses relating to its training, information dissemination and communication. Akudjedu et al. (2021:1221) reported 73.1% of their participants did not initially obtain any training on COVID-19, but after training, improvements were identified in their conduct in preventing the spread of infection.

Documents were analysed based on the WHO IPC document. Based on the results and analysis scale, the researcher developed; the assessed documents shared some similarities; some were deemed completely adequate based on the scale, while others were partially adequate. The hospital had a comprehensive document aligned with the WHO infection control guidelines. The results in the specific academic hospital showed that adherence to COVID-19 policies was not maintained as expected, and factors associated with environmental protocols lowered adherence when compared to direct care protocols. McBryde et al. (2020:57) report that policy development took place during the pandemic to assist healthcare workers in adopting new ways of functioning to prevent the spread of COVID-19. Based on the policy evaluation findings, the researcher identified that the academic hospital in Johannesburg had gaps in disseminating its policy, accessibility of the policy and lack of training; however, the assessment gave a fair indication of the policy’s availability and aspects covered therein (Mbunge 2020:1810).

#### Factor 1 – General protocols of coronavirus disease 2019

In this study, factors associated with environmental protocols had lower adherence than adherence to direct care protocols, and it is very important to make use of good environmental disinfection because it eradicates and reduces virus droplets (Pan et al. 2021:615). The study determined that nurses appropriately adhered to the COVID-19 policy when rendering direct patient care, where they had to be in direct contact with the patient. However, in general, nurses did not always show adherence towards the policy’s environmental protocols that included proper waste management, disinfection and management of the infectious environment. Medical wastes, such as needles and PPE, have a longer span of infection, and proper medical waste management is thus critical to prevent the spread of infection (Ilyas, Srivastava & Kim 2020).

#### Factor 2 – Direct patient care protocols for coronavirus disease 2019

Phase two’s questionnaire indicated that nurses scored their adherence under factor 2 high, which dealt with protocols to be applied before and after encountering the patient. However, when nurses were observed while carrying out their nursing duties, the researcher noticed a lower rate of adherence. A drop in hand hygiene practices, donning and doffing of gloves was observed in comparison to the self-administered adherence questionnaire (Purssell & Gould 2021:2065). In the absence of handwashing and for the sake of continuous nursing care and to prevent delays in emergency situations, alcohol-based hand gels can be used to prevent the spread of infection (Pan et al. 2021:615). Buheji and Buhaid (2020:19) suggest that greater emphasis on training on COVID-19 policies could help nurses provide care that does not put the nurse and patient at risk. This study assessed the availability of training, and the distribution varied; a high percentage of nurses mentioned having received training, while a significant number did not receive training.

#### Factor 3 – Rendering care to coronavirus disease 2019 patients

The third factor addressed one question: ‘Do you provide direct care to a suspected or confirmed COVID-19 patient?’. This meant that everyone coming to the hospital was treated as a person under investigation until their results were confirmed. Nurses needed to treat such persons as exposed patients and apply all relevant COVID-19 policy guidelines to prevent the spread of infection. Thus, nurses were often unsure if they were rendering nursing care to COVID-19 patients, potentially affecting nurses’ conduct and leading to non-adherence to the policy.

### Limitations

Data were collected from emergency departments, and these departments continuously received patients and provided care. This was a limitation because nurses in these departments were always busy, limiting the number of respondents who agreed to participate. The researcher could have been biased in observing the respondents and putting the Hawthorne effect in consideration because respondents could have changed behaviours upon seeing the observer. The validity of the instrument was confirmed through the pilot study, and stratified random sampling was used. The COVID-19 pandemic affected everyone and had an impact on nurses’ psychological state; this could have been a limitation, especially among nurses who lost someone because of Covid and could thus not participate. The study was conducted only in emergency departments, and nurses’ adherence to the COVID-19 policy in general departments was not considered. Therefore, the results are only based on the generalisation of emergency departments. Moreover, the study was conducted in a highly saturated COVID-19 area, which meant the researcher was at risk of contracting the virus in the process.

### Recommendations

Considering the study’s findings, recommendations are offered. Regarding nursing practice, nursing education, nursing research and nursing policy development specifically, several recommendations are made.

#### Nursing practice

Nursing is a profession governed by a scope of practice, guidelines, protocols and policies; therefore, stakeholders and management must always ensure the availability of nursing care-directing documents. A channel of communication should be designed to accommodate every nursing staff member. This includes mass e-mails, SMSs or daily updating messages in departments. Management should ensure in-service training is afforded to every nurse, especially on new aspects like the outbreak of a pandemic, because personnel will be dealing with it for the first time. This will also encourage a channel of direction to be followed when a pandemic occurs (Jingxia et al. 2022:7).

#### Nursing education

The curriculum should design strategic ways of dealing with pandemics, in a format to be followed by nursing students in the event of a pandemic to prevent interruption of learning, for example, adoption of online teaching and video teaching. The use of a simulation-based learning approach can be applied to reduce the risks associated with patient contact during a pandemic. The curriculum should include designing, disseminating and implementing policies early in the nursing qualification programme and continue during the programme to encourage student nurses to always function as guided by official documents like policies and guidelines.

#### Nursing research

A qualitative study can be conducted to determine why the actions of adherence were not maintained and to obtain nurses’ perceptions of COVID-19 and their experiences in the hospital. A study could be conducted with a larger sample to get a better generalisation of results. A study could be conducted to identify nurses’ adherence to infection control policies post-COVID-19.

#### Nursing policy development

Nursing policy development should not be limited to stakeholders and management but should engage nurses so that they contribute to the framework as they deal with direct patient contact. Policy development processes should also include the dissemination process tabulated in stages to reflect a direct objective that will cater for all personnel.

## Conclusion

The study explored nurses’ adherence to the COVID-19 policy while rendering nursing care to COVID-19 patients at an academic hospital in Johannesburg. Adherence was assessed, and gaps contributing to lower adherence were identified. The academic hospital in Johannesburg had a COVID-19 policy, but nurses indicated low accessibility, lack of dissemination and training on the policy, which the authors identified as adherence-limiting factors. The study further obtained conflicting results between the adherence phase and observation phase. In the adherence questionnaire, nurses scored their adherence to the policy high, but they scored low during the observation phase. This concludes that nurses were not fully adhering to the expected policy actions, and the study indicated a low adherence rate.
